# The remediation potential for PAHs of *Verbascum sinuatum* L. combined with an enhanced rhizosphere landscape: A full-scale mesocosm experiment

**DOI:** 10.1016/j.btre.2021.e00657

**Published:** 2021-06-27

**Authors:** Daniela Zuzolo, Rosaria Sciarrillo, Alessia Postiglione, Carmine Guarino

**Affiliations:** Department of Science and Technology, University of Sannio, via de Sanctis, Benevento 82100, Italy

**Keywords:** Polycyclic aromatic hydrocarbons, Microbial consortium treatment, *Verbascum sinuatum* L., Mesocosm experiment, Bioremediation

## Abstract

A full-scale mesocosm study was conducted to depict how integrated biological systems interact to adapt to contaminant stress and improve remediation of polycyclic aromatic hydrocarbons (PAHs)contaminated soils. The combination of *Verbascum sinuatum* L. and microbial consortium (fungi and bacteria) was employed along with three differently contaminated soils. After 240 days the highest PAHs removal (up to 68 %) and 6-rings compounds decrease was found in soil with lower pollution and cation exchange capacity. *V. sinuatum* showed a significant adaptability over time in terms of redox biology. Soil enzyme activities and microscopic evidences proved a rising plant-microorganisms association and a successful mycorrhization, arising from the inoculation of our consortia. In addition, an enhanced richness of PAHs degrading genes was achieved. Microbial co-metabolism, helped by the establishment of complex relationships with hosting plant, demonstrated to be suitable for the degradation of high molecular weight PAHs and represents a biotechnology with great prospects.

## Highlights

• AMF and bacteria enhance PAHs degradation patways.
• PAHs with 6 aromatic rings were drastically decreased.
• Our findings depict a successful mycorrhization.
• Soil enzymes activity increase over time.
• Our microbial consortia enhanced the presence of PAHs degrading genes.

## Graphical abstract

**Figure:**
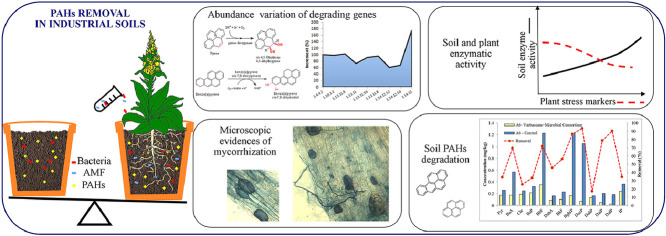

## 1 Introduction

Soil contamination may rise dramatic consequences for soil ecological function and concern about the potential health effects [1,2]. Hence, soil rehabilitation represents a worldwide challenge and is becoming a key-subject in post-industrial landscapes. Notably, polycyclic aromatic hydrocarbons (PAHs) are well known to be ubiquitous in soils, hardly degradable, which makes them one of the persistent contaminants in the environment especially at the soil level [3,4].

To date, biotechnological approaches inspired by nature such as phytoremediation are being explored to address the challenge of both removing PAHs from the soil and recovering soil ecological function. However, due to the low bioavailability of PAHs in soil, phytoremediation efficiency can be reduced in the practical application [5]. Several studies have shown that, combining phytoremediation and microbial remediation can be a promising tool to enhance the degradation/removal of PAHs [5,6].

For instance, the association of plants with microorganisms naturally occurring at the soil level have been successfully explored [7], [8], [9]. Generally, the rhizosphere zone has a strong physical and bio-chemical activities network influencing degradation mechanisms of xenobiotics [10]. In the context of rhizoremediation, plants have a non-secondary role since they colonize the contaminated space, shape the ideal rhizospheric environment and, consequently, the microbial structure, and function in the rhizosphere [11], [12], [13]. In general, different metabolites emitted by the plant as exudates are structurally similar to organic contaminants and these results in a microbial potential for degrading pollutants [12,14] and several enzymes released by roots act as a cometabolites to assist microbial organic compounds mineralization [15]. In addition, some organic acids emitted from the roots are able to facilitate the desorption of organic pollutants from soil particles making them more available for microbes [16]. The role of hosting horizontal gene transfer hotspots is generally recognized in the rhizosphere and plasmids have been shown to help microorganisms in adaptation to the stresses caused by contamination [17], [18], [19]. In this context, a successful rhizoremediation strictly depend on the selection of an appropriate plant genotype and on the soil bacterial community [20], [21], [22], [23] al.,. The role of bacteria in hydrocarbon degradation has been recognized by metatranscriptomics studies [11,24]. In addition, bacteria also play an important role as plant growth promoters that led to increased root extension and reduced stress for plants [25,26] . Another important tool in rhizoremediation is the role of Arbuscular Mycorrhizal Fungi (AMF), which are able to colonize the plant roots as well as create a remarkable hyphae extension exploring the surrounding environment [27]. The highly branched AMF mycelium can absorb nutrients beyond the root zone, providing a new path, the mycorrhizal path, for the absorption and transport of nutrients with low mobility. Fungi have a role for improving plant biomass growth and increasing stress tolerance, too [28], [29], [30]. In return, the plant offers carbohydrates to the fungi, which is a mandatory biotrophy. Many studies showed that ligninolytic fungi are particularly suitable for the degradation of recalcitrant PAHs [31], [32], [33]. Overall, the biodegradation pathways and associated genes have been researched and summarized in Biocatalysis/Biodegradation Database [16]. Therefore, a proper depiction of plant-microbe interactions aimed at rhizoremediation should consider the complex biological network facing with soil pollution in order to optimize its recovery [34]. The accumulation of enzymes at interfaces between soil and plant roots can be of great interest to investigate associations between plant and microorganisms [35]. Besides, in our opinion it is important the achievement of site-specific microbial consortia (to effectively degrade PAH mixtures) directly related to suitable native plant genotype. Among the recorded synanthropic flora in the Bagnoli brownfield site (Southern Italy), we observed the presence of *Verbascum sinuatum* L. [36], which frequently occur in disturbed lands of the Mediterranean area [37] since it is characterized by a great adaptation to unproductive soils.

With this in mind, the main aim of this full-scale mesocosm study was the assessment of the phytoremediation performance, on a long time frame, of *Verbascum sinuatum* L. and microbial consortium combination to cope with PAHs contaminated soil and successfully answer to remediation challenge. We designed the phytoremediation system at the metaorganism level rather than evaluating plants or microorganisms separately. The first specific objective include the investigation of the PAHs-degrading genes potential of the site, through specific metagenomic analyses of rhizospheric soils. The subsequent objective is the exploration of soil and plant enzyme activities to investigate (*i*) the propensity of the *Verbascum sinuatum* L. to accommodate the microbial consortium and (*ii*) the suitability to behave as a strengthened metaorganism able to remove PAHs from soil.

## 2 Material and methods

### 2.1 Plants material

In this study, *Verbascum sinuatum* L. (Ver) was assessed since it is a native species within the site and the genotypes used come from a selection of the observed phenotypes [36]. The choice of *V. sinuatum* (biennial cycle plants with buds placed at ground level) was based on a previous study carried out on the site concerning the analysis of all the genotypes present in the area and their rhizospheric effects concerning the degradation of hydrocarbons . Metagenomics studies have revealed a rhizospheric bacterial diversity associated with this very interesting species and the occurrence of numerous PAHs-degrading genes [6]. The *V. sinuatum* plant develops a substantial biomass and is extremely resistant to abiotic stress [37]. As matter of fact, the Bagnoli brownfield site shows a consistent *V. sinuatum* vegetal cover. The seeds collected come from phenotypes that showed a better plant structure (specific biomass).

### 2.2 Consortium used

The microbial consortium was selected in previous research about Bagnoli site [6] in which both native PGPR and PAHs degrading microorganisms were found. It is named “Consortium Bagnoli 2018@” and conservated in the bank of microorganisms particularly suitable for xenobiotic conditions owned by the Department of Science and Technology of University of Sannio (Italy). It is composed by: *Rizophagus clarum, Rizophagus intraradicens, Rizophagus irregularis, Rizophagus proliferus, Glomus macrocarpum, Glomus spp, Claraideoglomus claroideum, Claraideoglomus eutinicatum, Gigaspora marginata, Gigaspora gigantea, Acaulospora spp, Burkholderia gladioli, Burkholderia cepacica, Pseudomonas putida, Pseudomonas fluorescens, Pseudomonas spp*., *Comamonas koreensis, Serratia proteamaculans, Bacillus cereus, Bacillus licheniformis, Bacillus megaterium, Bacillus polymyxa, Bacillus subtilis, Bacillus thuringiensis, Paenibacillus polymyxa* and *Pleurotus ostreatus*.

### 2.3 Experimental design

The experiments was carried outdoors on the Bagnoli brownfield area falling into the western part of the city of Naples (Southern Italy) from October 2018 to July 2019 (240 days). This site is one of the main dismissed industrial areas in Italy, with a long history of pollution, mainly PAHs related [6,36,[38], [39], [40]]. Soil was excavated from three different areas (A_3_, A_4_ and A_6_) of the site with diverse physico-chemical features (Table S1 – Supplementary Materials). Three composite topsoil samples (0-30 cm depth) were collected at each area using a soil spade and stored to form the mesocosms. In total, 30 mesocosms (10 replicates for each area) were designed with about 29 kg of contaminated soil of A_3_, A_4_ and A_6_ areas. The soils of the mesocosms were manured with mineral and organic mixture composed by Ammonium sulphate (150g), Ammonium phosphate (150) and organic substance (150 g) with inorganic NPK fertilizing elements. Besides, each pot was added 20g of “Consortium Bagnoli 2018@” (485 million of spores for kg of soil). In addition, 300 g of hay bale inoculated with *Pleurotus ostreatus* (Jacq.) P. Kumm., Führer Pilzk. (Zwickau) was added.

Subsequently, twenty seeds (20) of *V. sinuatum* were introduced in mesocosm. To monitoring the bioremediation processes, soil were collected at the beginning of the experiment (T_0_) and after 60 days (T_1_-December 15^th^ 2018), 120 days (T_2_-March 15^th^ 2019) and 240 days (T_F_-July 15^th^ 2019) whereas plant tissues (roots and leaves) were collected after 60 days (T_1_-December 15^th^ 2018), 120 days (T_2_-March 15^th^ 2019) and 240 days (T_F_-July 15^th^ 2019) and immediately frozen (−80°C) until analysis. In addition composite soil samples (representative of the three areas mesocosm soil) were collected before (without the microbial treatment) and after the experimental trial for functional metagenomic analyses.

### 2.4 Analytical methods

#### 2.4.1 Soil and plant (roots and leaves) PAHs analyses

For this study, the target analytes were the 4, 5 and 6 rings US EPA priority PAH compounds: Pyrene (Pyr), Benzo[a]anthracene (BaA), Chrysene (Chr), Benzo[a]pyrene (BaP), Benzo[b]fluoranthene (BbF), Dibenzo[a,h]anthracene (DahA), Benzo[k]fluorantene (BkF), Benzo[g,h,i]perylene (BghiP), Dibenzo[a,e]pyrene (DaeP), Dibenzo[a,h]pyrene (DahP), Dibenzo[a,i]pyrene (DaiP), Dibenzo[a,l]pyrene (DalP), Indeno [1,2,3-c,d] pyrene (IP).

The analysis of PAHs in soil and plant material followed the procedure described in [6]. Ten grams of sieved (2 mm fraction) soil sample was weighed, spiked with PAH surrogates and extracted with dichloromethane (CH_2_Cl_2_). A alumina/silica gel column was used to clean up the extracts. The eluates were then concentrated under a gentle stream of nitrogen and analyzed by GC-MS (7890 A, Agilent, USA). Deuterated PAH surrogate (Deuterated fluorene, Deuterated fenanthrene, Deuterated chrysene and Deuterated perylene) standards were added to the samples to monitor the procedures of sample extraction, treatment and analysis. For plant analysis two grams of each sample (roots and leaves) were pulverized with sodium sulfate anhydrous (Na_2_SO_4_) by a ceramic mortar and pestle to remove excess moisture. Then pulverized samples were extracted, cleaned and analyzed as above described.

In order to ensure the validity of the analyses during the experiment, different control procedures were adopted. At every 10 samples were enclosed the determination of a certified reference material (Beechwood (PCP and PAH) BCR® certified Reference Material- Sigma-Aldrich, Italy) such as quality control. Blanks and matrices plus standard addition (a mixture of 13 EPA PAHs and 4 Deuterated PAHs) were quantified sporadically to determine the accuracy of the testing. Procedural blank samples were quantified sporadically to check for cross-contamination. The target PAHs compounds were not detectable in the solvent blank samples.

#### 2.4.2 Soil enzyme activity analysis

The polyphenol oxidase (PPO) activity, the dehydrogenase (DHO) activity, the urease (URE) activity and the alkaline phosphatase (ALP) activity and the catalase (CAT) activity were assayed in triplicate air-dried samples. For PPO activity assessment, 1 g of soil sample was mixed with 10 mL of 1.0% pyrogallol solution for 2 hours at 30°C. Citric acid-phosphate buffer with ether was added to the solution for 30 minutes. The ether was colorimetrically measured (430 nm). The control tests without soil and without pyrogallol were simultaneously performed [41]. For DHO, 0.03 g of CaCO_3_ and 0.5 mL of 3% triphenyl tetrazolium chloride (TTC) were mixed with soil sample and incubated at 37°C in the dark for 24 hours. After adding 5 mL of methanol, the solution was filtered using a glass funnel capped with absorbent cotton until no red color remained. The samples were determined colorimetrically (485 nm) after being diluted with methanol. Control assays (without CaCO_3_ and TTC) were simultaneously conducted [42]. For URE activity evaluation, a solution of toluene, citrate buffer and urea were mixed with soil and subsequently incubated at 37°C for 24 hours. The produced ammonium was determined colorimetrically (578 nm) using the blue Indophenol. A control (without urea) was used for each sample [41]. For ALP activity, the soil was mixed with disodium phenylphosphate and borate buffer and incubated at 37°C for 2 hours. Potassium hexacyanoferrate in alkaline solution was used to extract and oxidize the phenol produced. Measures were done by the 4-aminoantipyrin colorimetric method at 510 nm. Tests without soil and disodiphenyl phosphate were assessed as controls [41]. The catalase activity was determined through the reduction of potassium permanganate (KMnO4) by hydrogen peroxide. A soil sample was mixed with a 3% hydrogen peroxide solution and H_2_SO_4_, shaked for 20 min and the filtrate was titrated with 0.1 mol/l KMnO4. Catalase activity was expressed as ml 0.1 mol/l KMnO4/g/h [43].

#### 2.4.3 Metagenomic sequencing library preparation

Functional gene analysis based on metagenomic sequencing was performed for soil before and after the experimental trial to depict the quantitative variation of genes directly involved in PAHs degradation and other metabolic pathways. Library and the sequencing of the genes was performed following the method of [6,9]. The “Ovation® Ultralow Library System V2” kit (Nugen, San Carlos, CA) was used for producing library. The samples were quantified and quality tested using the Qubit 2.0 Fluorometer (Invitrogen, Carlsbad, CA) and Agilent 2100 Bioanalyzer (Agilent Technologies, Santa Clara, CA). Libraries were processed and sequenced on MiSeq (Illumina, San Diego, CA), pair-end with 300 cycles per read. Base calling and demultiplexing were performed on instrument. The sequencing run produced 0.91 M and 1.15 M of reads (in millions) for the two sample types (before and after the experimental trial), respectively.Gene sequences involved in PAHs degradation were obtained from Uniprot, and the short reads resulted from the sequencing experiments were blasted against the above-mentioned genes using BLASTX v2.2.29. All reads significantly matching (with an e-value lower than 0.1) were retained for analysis.

#### 2.4.4 Plant stress markers and antioxidant enzyme

Thirty fresh leaves (50 g) of *V. sinuatum* were homogenized under liquid nitrogen into a fine powder and were centrifuged at 19,000 g for 30min at 4°C. The activity of stress marker and antioxidant enzymes such as Glutathione S-Transferase (GST), Phenylalanine Ammonia Lyase (PAL), Proline and Lipid peroxidation (MDA), (Superoxide dismutase (SOD), Catalase (CAT), Ascorbate peroxidase (APX), Guaiacol peroxidase (GPX) were assessed as described by [25,42] since they're natural biomarkers to measure the abiotic stress in plants [44].

#### 2.4.5 Experimental evidence of mycorrhizal colonization

The roots sampled at 60 and 240 days after seeds planting were used to observe the development of host root colonization over time. In addition, roots of spontaneous adult *Verbascum sinuatum* L. grown in-situ were sampled to depict the inherent mycorrhizal potential.

The fine roots were fixed in FAA fixative solution (10% Formalin: 5% acetic acid: 50% ethanol: 35% deionized water). The fixed roots where then processed with the staining procedures described by [45] with the modifications applied by [46]. The roots were cleared in 10 % KOH at 121°C for 15 min to remove the host cytoplasm, most of the nuclei and pigments, rinsed in water, acidified for 20 s in 3 % HCl, rinsed in water, and then stained in 0.05 % Trypan blue solution in lactoglycerol (lactic acid/glycerol/deionized water in a mixing ratio of 1:1:3) for 5 minutes. The excess stain was removed in clear lactoglycerol at room temperature. Roots are then placed on glass slides for microscopic observation. The percentage of intracellular hyphal colonization was assessed using a Nikon Eclipse E600 microscope.

Magnified Intersection Method [47]was used to score, on an objective scale of measurement, differential mycorrhizal colonization between samples. A number of nine *Verbascum sinuatum* plants (three from mesocosm system at T_1_, three from mesocosm system at T_F_ and three from the field) were taken. Roots from each plant were sampled and divided into 5 subsamples. Each subsample included 12 fragments of approximately 1 cm length. Root fragments stained with the procedures described above [45] were aligned along the long axis of the slide and observed under light microscopy with 200x magnification. The field of view of the microscope was moved using the stage to make eight constant passes across each fragments to obtain perpendicular intersection of the vertical crosshair with the root. Ninety-six intersections per subsamples were analyzed. Every time that the vertical crosshair intersection cut any fungal structure (such as arbuscule, vesicle or hyphae) the annotation increased of one. Each subsamples was scored three times using shifted starting observation point. Arbuscular Colonization (AC), Vesicle Colonization (VC) and Hyphal Colonization (HC) were calculated as reported by [47] and expressed in percentage.

#### 2.4.6 Data analyses

The data presented are the mean ± SE. Phytoremediation performance was calculated for each analyzed PAH compound as follows [48]:

$$\text{Removal}\;\text{performance}\;\left(\%\right)=\frac{C_{T_{0}}-C_{T_{F}}}{C_{T0}}x\;100$$

where $C_{T_{0}}$ and $C_{T_{F}}$ are the initial and final PAH compound concentration in soil.

The data were statistically analyzed by means comparison by the parametric ANOVA and the Tukey's HSD (honestly significant difference) procedure was adopted for pairwise comparisons. Statistical analyses were conducted to (i) assess whether soil type may have influenced PAHs removal rate, (ii) to test the influence of soil type and time in soil enzyme activity, (iii) to evaluate whether plant antioxidant response significantly differ over time. The statistical significance was verified at a level of *P* < 0.05. The *Stats* package in R environment [49] was used for statistical data analyses.

## 3 Results

### 3.1 PAHs degradation and removal performance in different soil mesocosms

**Fig. 1:**
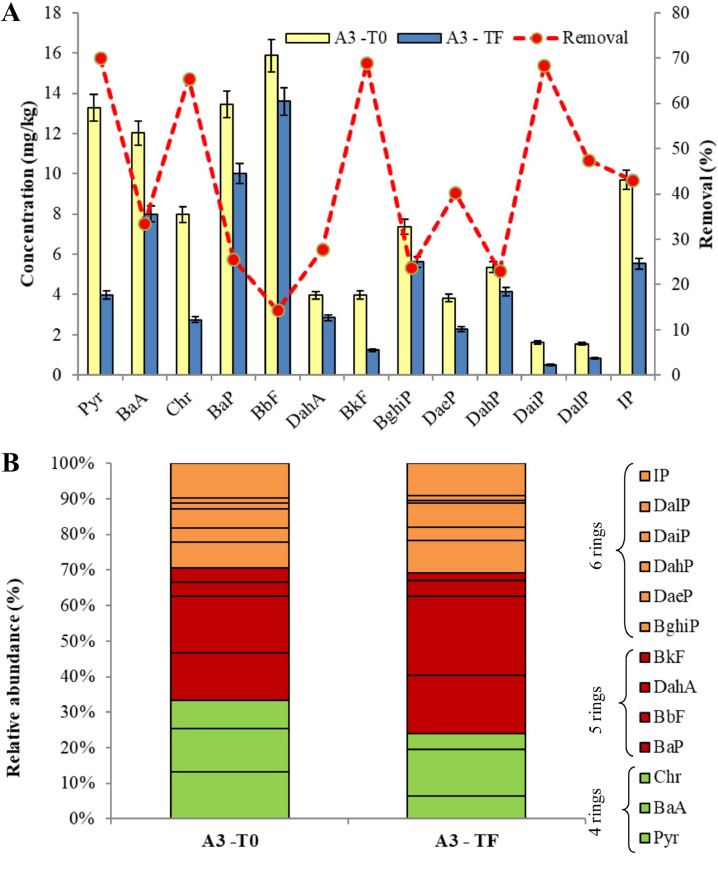
Comparison of PAHs pattern of A_3_ soil: (A) concentration levels at the beginning (T_0_) and at the end (T_F_) and removal performance of the phytoremediation system; (B) congener's profiles of PAHs at the beginning (T_0_) and at the end (T_F_).

**Fig. 2:**
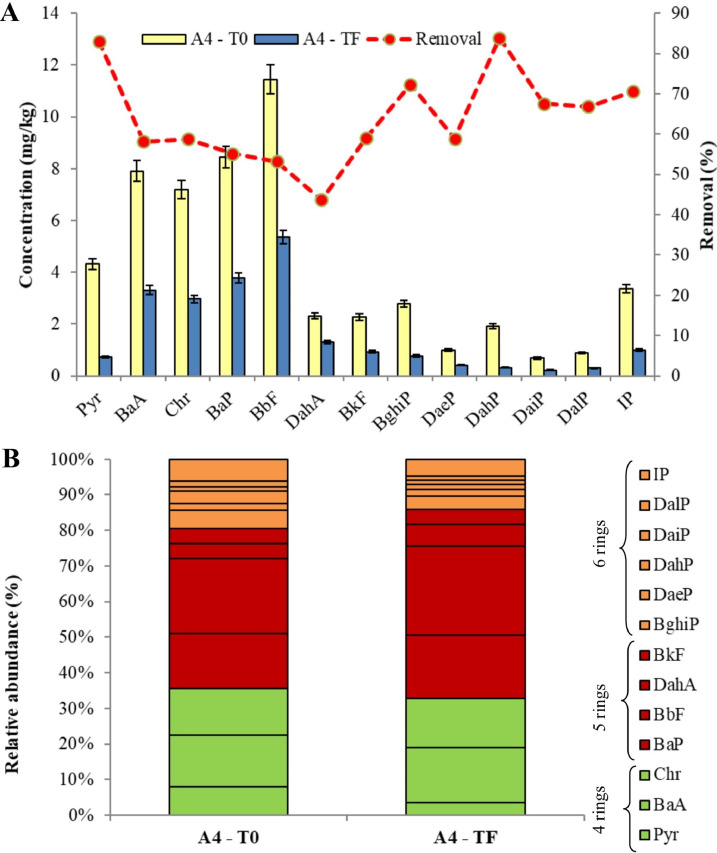
Comparison of PAHs pattern of A_4_ soil: (A) concentration levels at the beginning (T_0_) and at the end (T_F_) and removal performance of the phytoremediation system; (B) congener's profiles of PAHs at the beginning (T_0_) and at the end (T_F_).

**Fig. 3:**
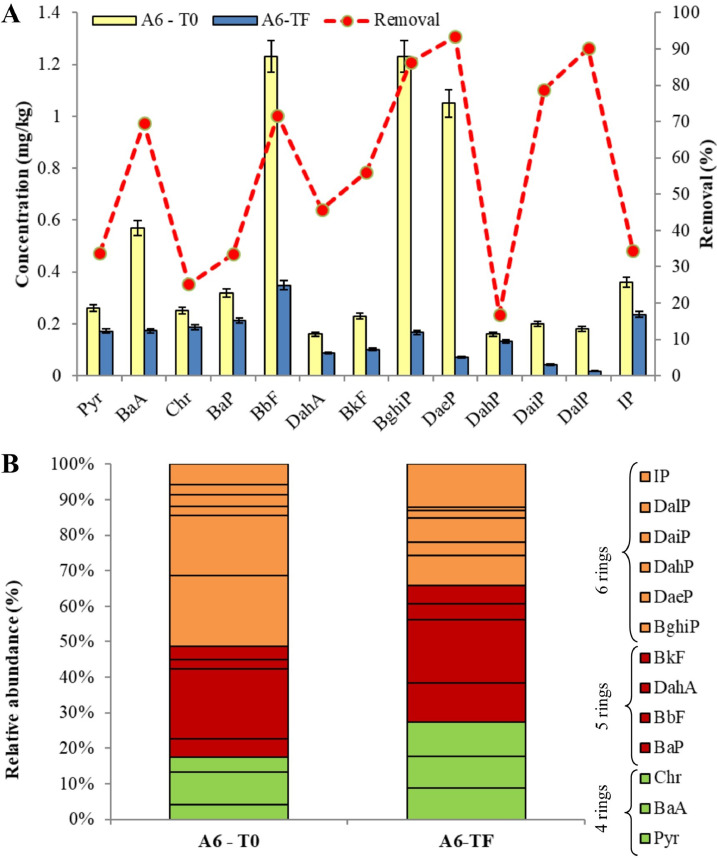
Comparison of PAHs pattern of A_6_ soil: (A) concentration levels at the beginning (T_0_) and at the end (T_F_) and removal performance of the phytoremediation system; (B) congener's profiles of PAHs at the beginning (T_0_) and at the end (T_F_).

Fig. 1, Fig. 2, Fig. 3 show the degradation of the different PAHs congeners into the soil of treated mesocosms. A valuable PAHs decrease in all the three investigated soil (A_3_, A_4_ and A_6_) was observed after 240 days.

The ANOVA results (considering F statistics) showed that removal rate were significantly different between the soil types. Tukey's HSD test allowed us to determine that PAH removal in A_3_ soil was significant different from A_4_ and A_6_ ones at *p < 0.05*.

The total PAHs concentrations in A_3_ soil at T_0_ was 99.9mg/kg, while the final concentration decrease to 61.3 mg/kg, showing an overall removal of 38.7%. After the experimental time, less than 35% of Pyr, Chr, BkF and DaiP were remaining in the A_3_ mesocosm soil (Fig. 1A). For instance, initial Pyr concentration was 13.3 mg/kg, which declined to 3.98 mg/kg. The different congener's profiles of PAHs (Fig. 1B) in soil indicated that the 4-rings compounds (Pyr, BaA and Chr) mostly contributed to total concentration of PAHs at the beginning of the experiment. Our treatment enhanced the degradation of 4-rings compounds highlighting that 70%, 33% and 65% of Pyr, BaA and Chr were respectively degraded after 240 days.

As regards A_4_ soil, after a 240-day period, there was an outstanding removal rate of total PAHs in the soil (60.8%), with total PAHs concentration level decreasing from 54.5 to 21.4 mg/kg.. The highest removal rates (Fig. 2A) were recorded for Pyr and DahP, with more than 83%, decreasing from an initial concentration of 4.32 and 1.93 mg/kg to a final one of 0.73 mg/kg and 0.31 mg/kg (Pyr and DahP, respectively). Therefore, according to Fig. 2B we noticed that A_3_ subzone showed a better removal rate for 4-rings and 6-rings PAHs.

Relative to A_6_ soil, the degradation rate of ΣPAHs was up to 68% (from 6.2 to 1.9 mg/kg), and the soil content of PAHs with 6 aromatic rings (BghiP, DaeP and DalP) was drastically decreased (Fig. 3B). A_6_ soil reported the lowest degradation degree of Pyr and Chr (4-ring PAHs) with 34% and 25% of removal rate. Our findings showed an enhanced PAHs degradation in soil with lower levels of pollutants, such as A_4_ and A_6_ areas. However, also cation exchange capacity (CEC) of soil particles, which was lower in such soils (Table S1 in Supplementary Materials), may have had contribute to higher bioavailability of PAHs and, thereby, to their removal [50]. On the contrary, the much higher TOC of A_3_ soil could inversely affect PAHs availability [51].

Overall, the enhanced microbial degradation pathways could mainly explain the PAHs decrease in soil. As matter of fact, it has been observed that PAHs plant uptake is quite negligible as shown in Tables S2 and S3 in Supplementary Materials (in accordance to previous studies, e.g., [52]).

### 3.2 Microscopic evidences of arbuscular mycorrhizal colonization: structures and developmental stages

A certain mycorrhizal colonization rate was observed in adult Verbascum roots sampled from the field due to the native soil microorganisms (Table S4 in Supplementary material). As regards plants grown in mesocosm experiment, the percentage of root length colonized by vesicles, arbuscules and hyphae varied between the two experimental times (Table S4 in Supplementary material). Mycorrhizal colonization is already manifest at 60 days and showed an Arbuscular Colonization (AC), Vesicle Colonization (VC) and Hyphal Colonization (HC) of 3.4%, 6.6% and 26.1%, respectively. However, AC, VC and HC increased up to 12.1%, 16.5% and 49.7% (respectively) at the end of our experiment (240 days). The most interesting outcome is surely the one of AC which is 4 times higher at the end of mesocosm experiment, demonstrating that a successful mycorrhization, arising from the inoculation of our consortia, was achieved and that it is an active and progressive process.

**Fig. 4:**
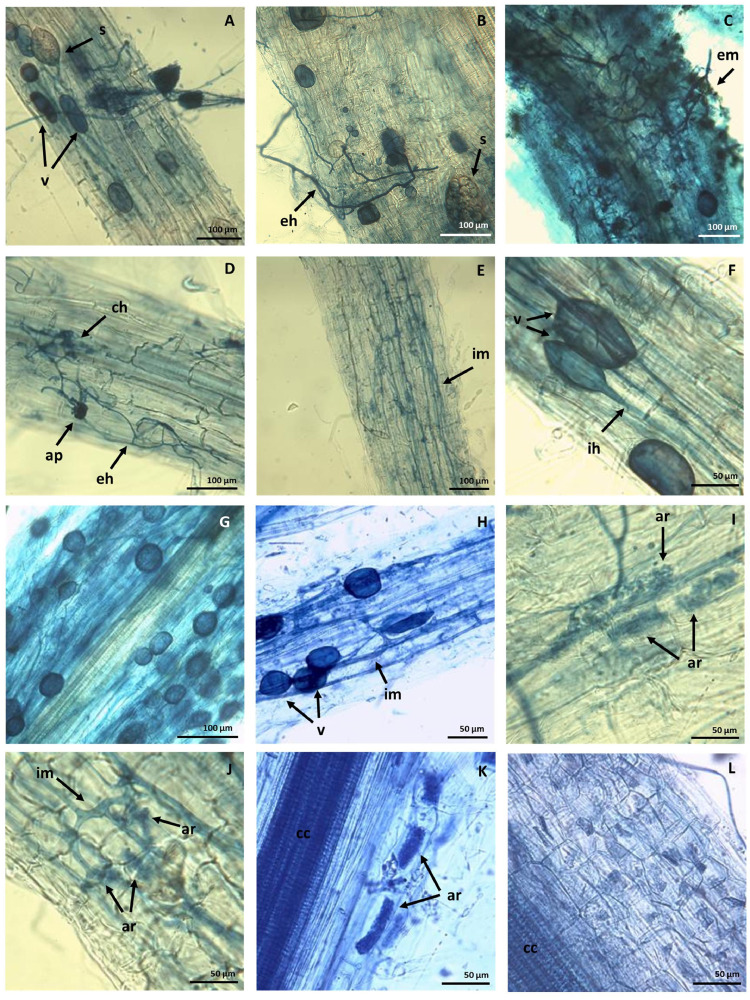
Mycorrhization of *Verbascum sinuatum* L. grown in mesocosm experiment (sampled at T_F_). Roots are detected by Trypan Blue staining and light microscopy (using Vohník et al., 2016 protocol). (A) Intracellular vescicles (v)and extracellular spores (s); (B) external hypae (eh) and extraradical spores (s); (C) external hypae forming an extraradical mycelium (em); (D) external hypae (eh), entry point with appressorium (ap) formation, coiled hypae (ch); (E) intraradical hypae running through, parallel to the root axis and spreading forming an intraradical mycelium (im); (F) vesicles (v) and internal hypae (ih); (G) roots completely filled up with vesicles (v); (H)vescicles and intraradical mycelium (im); (I–L) *Arbuscular mycorrhiza* (ar) in root fragments often completely filling the root cells; central cilinder (cc).

A continuous mycorrhization was depicted by the microscopic examination. In Fig. 4A and B external VAM spores (s) are visible. Mycorrhizal associations may be initiated by spore germination that form an extraradical mycelium (em) (Fig. 4C), whose hyphae (eh) (Fig. 4D), are responding to the presence of a root by growing towards it, establishing contact and growing along its surface.

Then, one or more hyphae produce swellings called appressorium between epidermal cells and penetrate amongst and within cortical cells of the root forming an intraradical mycelium (im) (Fig. 4E). Fig. 4C highlights the complex hyphae structure that colonizes the root of *Verbascum* from outside. Fig. 4D depicts germinative external hyphae (eh) contacting suitable roots (not lignified yet) and shaping the appressorium. Fig. 4E shows these hyphae cross the hypodermis (through passage cells if these are present in an exodermis) and start branching in the outer cortex. The coiling of these hyphae (ch) in cortical cells indicates the penetration of these infected hyphae. The vesicles (v) structures (showed in Fig. 4F) are terminal hyphal swellings, which may be inter-or intracellular and have a storage function [53]. Fig. 4G and H show root sections filled by vesicles, interconnected by internal mycelium. Arbuscles (ar), instead, are the intracellular structure interfacing for the nutritional exchanges between fungus and hosting plant [54]. Our findings highlight that arbuscular mycorrhizae completely fill the root cells of *Verbascum* (Fig. 4K). Different root section showed different degrees of vesicles, Arbuscles and hyphae abundance. However, or results showed that colonization never crosses the perycicle and never reaches central cylinder (cc) of the roots but it is limited to root cortex.

### 3.3 Soil and plant enzymatic activity relationship

Our data about the variation in PPO, DHO, ALP and URE and CAT activities in rhizosphere soil show their increase over time (Tables 1 and 2), which may suggest a highly consolidated associations between plant and microorganisms [9,35,42]. Statistical analyses showed that soil enzymatic activity does not significantly (at *p < 0.05*) vary among the three experimental plot (A3, A4 and A6) but depend on time.

**Table 1:** Enzymatic activities (DHO, PPO, ALP, URE and CAT) in mesocosm soil of three subareas (A3, A4 and A6) at different times: T_0_, 60 days (T_1_), 120 days (T_2_) and 240 days (T_F_). Mean (3 replicates) ± ES is shown. Different letters indicate significant difference between experimental times at the level of P < 0.05.

Sample	DHO (µgTPFg-1min-1)	PPO [m/(mg g-1soil 2h-1)]	ALP [m(phenol)/(mg g-1soil 24h-1)]	URE [mNH3-N)/(mg g-1soil 24h-1)]	CAT (ml 0.1 mol/l KMnO4/g/h)
A3_T0	9.1±0.05^a^	0.18±0.01^a^	0.023±0.001^a^	0.33±0.02^a^	0.89±0.05^a^
A3_T1	11.9±0.08^b^	0.21±0.02^ab^	0.026±0.001^b^	0.39±0.01^b^	1.1±0.07^b^
A3_T2	13.1±0.06^b^	0.29±0.01^b^	0.028±0.001^bc^	0.41±0.02^b^	2.41±0.06^c^
A3_Tf	14.6±0.08^b^	0.28±0.01^b^	0.029±0.001^c^	0.44±0.03^b^	2.44±0.05^c^
A4_T0	7.5±0.05^a^	0.22±0.02^a^	0.021±0.001^a^	0.28±0.01^a^	0.78±0.01^a^
A4_T1	10.3±0.06^b^	0.27±0.01^ab^	0.026±0.001^b^	0.32±0.02^b^	1.32±0.04^b^
A4_T2	12.2±0.05^b^	0.26±0.01^b^	0.027±0.001^bc^	0.37±0.05^b^	2.37±0.03^c^
A4_Tf	15±0.04^b^	0.23±0.02^b^	0.028±0.0001^c^	0.4±0.05^b^	3.4±0.01^c^
A6_T0	7.1±0.02^a^	0.17±0.01^a^	0.019±0.001^a^	0.32±0.01^a^	0.99±0.01^a^
A6_T1	9.2±0.04^b^	0.18±0.01^ab^	0.024±0.001^b^	0.34±0.02^b^	2.34±0.05^b^
A6_T2	10.4±0.05^b^	0.21±0.05^b^	0.026±0.001^bc^	0.39±0.0^b^	2.39±0.03^c^
A6_Tf	12.7±0.08^b^	0.28±0.01^b^	0.026±0.001^c^	0.41±0.01^b^	2.41±0.02^c^

**Table 2:** Activity of SOD, CAT, GPX and APX, GST, PAL, Proline content and MDA in leaves of *Verbascum sinuatum* L. at different times: 60 days (T_1_), 120 days (T_2_) and 240 days (T_F_). Mean (3 replicates) ± ES is shown. Different letters indicate significant difference between experimental times at the level of P < 0.05.

Sample	SOD (Umg/protein)	CAT (nmol H2O2 mg−1 protein min−1)	APX (nmol ascorbate mg−1 protein min−1)	GPX (nmol guaiacol mg−1 protein min−1)	GST (µM/min/µg protein)	PAL (µg t-cinnamic acid/h/µg protein)	PROLINE content (µmol g fw)	MDA (µmol/g fw)
A3_T_1_	88.45±2.50^a^	141.12±9.0^a^	258.48±15^a^	81.19±2.90^a^	101.87±3.5^a^	89.88±2^a^	258.98±2.5^a^	106.46±5^a^
A3_T_2_	75.46±2.66 ^ab^	128.34±8^ab^	236.51±7.0^ab^	75±3.50^ab^	93.28±1.50^a^	81.35±1.90^ab^	256.47±2.9^a^	104.37±4^ab^
A3_T_F_	61.11±3.01^b^*	112.65±7.0^b^	187.25±5.0^b^	70±4.50^b^	81.26±2.30^b^	78.27±3.0^b^	222.87±2.5^b^	101.38±2.5^b^
A4_T_1_	44.23±1.51^a^	106.14±10^a^	210.16±10^a^	79±6.50^a^	116.34±2.0^a^	33.56±1.78^a^	233.45±11^a^	81.23±2^a^
A4_T_2_	40.21±1.01^ab^	98.65±5.6^ab^	196.37±4.9^ab^	75±4.0^ab^	111.47±2.5^a^	33.21±1.55^ab^	221.47±9^a^	79.27±2^ab^
A4_T_F_	36.54±1.11^b^	95.56±4.50^b^	176.26±3.9^b^	59±2.50^b^	89.46±1.5^b^	30.26±1.70^b^	210.01±8^b^	76.82±3^b^
A6_T_1_	64.14±1.90^a^	123.14±10^a^	219.38±10^a^	65±2.50^a^	101.35±10^a^	28.45±1.90^a^	248.56±11^a^	86.45±4^a^
A6_T_2_	53.11±1.78^ab^	112.5±11^ab^	233.26±11^ab^	56±2.0^ab^	98.45±3.5^a^	27.46±1.55^ab^	244.59±9.5^a^	82.48±3a^b^
A6_T_F_	51.13±1.23^b^	103.45±10^b^	220.49±15^b^	51±2.0^b^	85.31±3.0^b^	25.28±1.45^b^	220.37±2.5^b^	78.26±3.5^b^

The soil alkaline phosphatase (ALP) in soil, which originates from soil bacteria and fungi [55] v, showed a rising trend in all soil (A3, A4 and A6), with a higher value for A3 soil. ALP at T_0_ is significantly different from that at 60, 120 and 240 days (Table 1). It is an indicator of the potential bioactivity of soils and is closely related to the level of fungal colonization in roots [56]. In addition, ALP may be connected to active P assimilation or transport in mycorrhizal roots [57].

The activity trend of urease (URE) showed a significant increase after T_0_. The amount of URE activity in soil is correlated with the microbial activity [58]. It is involved in the hydrolysis of urea to ammonia and carbon dioxide it is a biological indicator in the nitrogen cycle [59]. Both ALP and URE data indicate that a potential recovery of the ecosystem function of soils, since they have an important role in nutrient cycling.

During the period of the experiment, DHO activity in the soil increased up to 2 times, with a significant increase in the first 60 days. This behavior may be due to the DHO role in the degradation of high-ring PAHs during the initial period to induce hydrogen segregation, which acted as increased DHO activity in the experiment (as also suggested by [41,60]).

In addition, CAT activity significantly rise after 60 and 120 days in the three type of soils at *p < 0.05*. The Tukey post-hoc results also indicate that CAT activity remains almost unchanged from 120 days until the end of the experimental time. CAT in soil (synthesized by microorganisms) is able to prevent the toxic effects of oxidative stress caused by PAHs, splitting hydrogen peroxide into oxygen and water molecules. In addition, the proliferation of soil microorganisms can cause an increase in the activity of CAT.

Regarding the polyphenol oxidase (PPO), the post-hoc multiple comparison test revealed that minimum activity levels were recorded at T_0_ and the higher PPO levels were observed at 120 and 240 days. Our results showed about the plant antioxidant response are presented in Table 2.

Proline (which is a crucial osmoprotectant) data indicated a significant decrease at 240 days (T_F_). Its content in leaves under environmental stress is of absolute relevance for plant adaptation [61]. Our results showed that MDA levels during the earliest experimental stage are significantly higher (due to strong plant internal detoxification mechanisms) than those at 240 days (T_F_). Our findings are in accordance with other researches [25,62] and may suggest that the plants required some period to tolerate the contamination. Phenylalanine Ammonia Lyase (PAL) play an important role in plant development and is a key enzyme in plant stress response since it's biosynthesized under oxidative stress [63]. As matter of fact, our data about stress markers showed a downward trend leading to a significant reduction of MDA, Proline and PAL at T_F_.

### 3.4 Metagenomic insights: co-metabolism of microbial consortium

**Fig. 5:**
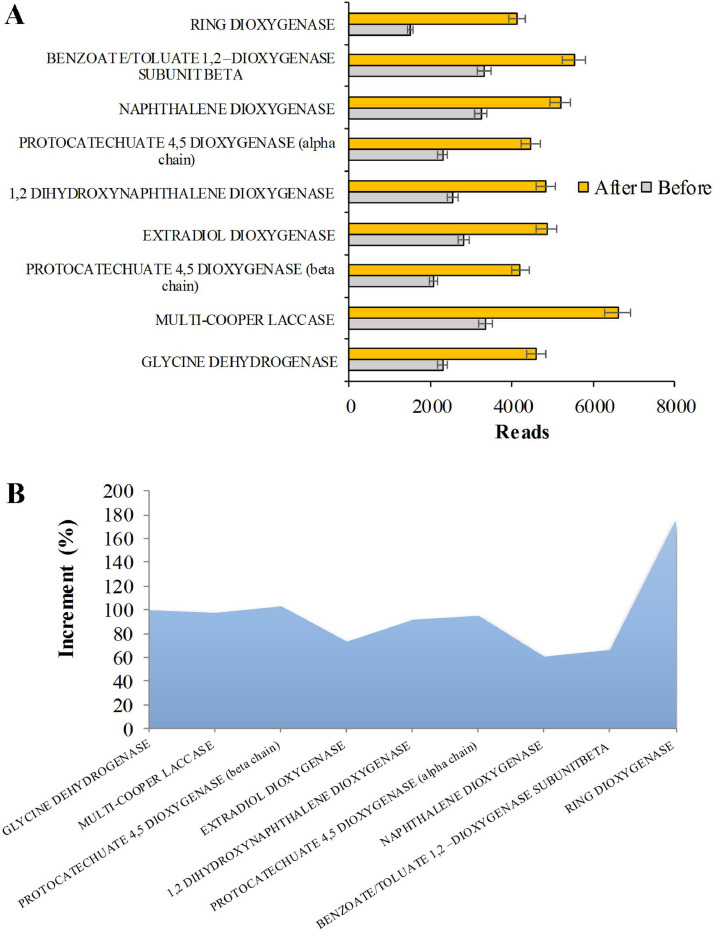
PAHs degrading genes from soil metagenomes. (A) Abundance of PAHs degrading genes before and after the experiment. (B) Relative increment of observed gene encoding for PAHs degradation.

The PAHs degradation pathway is a very complex phenomenon. It can be assumed that PAH degradation is the outcome of co-metabolism mediated by fungi and bacteria, with the fungi involved in the earliest oxidation step [64,65]. Our data about the genes encoding enzymes for PAHs degradation of soil without the microbial treatment depict the inherent natural soil microorganism populations capable to use specific enzymes to attack PAHs (Fig. 5A). However, the addition of our microbial consortia generally enhance the presence of PAHs degrading genes. Among them, laccase is the most abundant class. Laccase (EC 1.10.3.2), also named phenolase, are multi-copper oxidase widely found in nature and occur in plant, fungi and bacteria. However, fungi are the major laccase producers [66]. It is well known that filamentous fungi are able to secrete extracellular laccase enzymes that are able to break the chemical bonds of PAHs compounds through oxidation of recalcitrant PAHs. Laccase transform PAHs immediately upon entering the soil [67]. However, due to lack of suitable enzymes, generally fungi are not able to totally degrade high molecular weight PAHs, but can transform them into polar metabolite(s) with their extracellular enzymes, which can further be degraded by bacteria and other microbes. Our results also highlight that also bacteria play a key role in the initial PAHs degrading steps. As matter of fact, the higher increase (after the microbial consortium addition) was observed for gene encoding ring- dioxygenases (EC 1.14.12) as shown in Fig. 5A and B.

Besides, also gene related to estradiol ring-cleaving dioxygenases were double compared to the soil without microbial consortia. Aromatic hydrocarbon dioxygenases have been reported to catalyze the initial reaction in the bacterial biodegradation of a diverse array of aromatic and polyaromatic hydrocarbons. These enzymes are cofactor-requiring multicomponent heteromultimeric proteins that catalyze the initial activation through reductive dihydroxylation of their substrates, and are distinct from aromatic ring-cleavage (or ring-fission) dioxygenases (EC 1.13.11) which act on catechol intermediates in many of the same catabolic pathway. Ring-dioxygenases act adding both atoms of O_2_ to the aromatic ring of the compound (dihydroxylation), with the formation of a *cis*-dihydrodiol, which is converted to a diol intermediate by the action of a dehydrogenase. The next step in the degradation is ring fission by means of cleavage by intradiol or estradiol ring-cleaving dioxygenases (EC 1.13.11) which lead to intermediates such as catechols. After this ring fission, several reactions can occur until turning catechols into TCA cycle intermediates [65,68]. Finally, the intrinsic PAHs degrading ability of naturally occurring microbial consortia in the studied industrial soil seems to be enhanced by the improvement of the microbial consortia. Co-metabolism of fungi and bacteria, helped by the establishment of complex relationships with hosting plant, can intensify the synergistic effect especially for the degradation of HMW PAHs [65,69].

## 4 Discussion

*Verbascum* specie was chosen since it is well adapted to the industrial soil under investigation, it can tolerate xenobiotic stresses and above all, it developed cooperative adaptation with associated microbiota, leading to the existence of naturally occurring well adapted metaorganism [6,36,39]. The selection of the genotype is of fundamental importance in the rhizosphere engineering and must be based on the "natural" ability of the chosen plant species (*i*) to grow-up the contaminated soil, (ii) to develop a wider and deeper root exploration, (*iii*) to be inclined for endo-ectophytic relationships with microorganisms and (*iv*) to produce radical exudates for the development of signals and co-evolutionary relationships. In addition, other authors underlined the relevance of plant choice in order to promote and accelerate remediation of hydrocarbon-polluted lands [34,70]. Therefore, the combination of the genotype and useful microbial consortium is not trivial and must be based on the in-depth study rhizosphere environment of the chosen plant.

Our findings within this full scale mesocosm experiment have revealed important features of the studied metaorganism in the scope of biodegradation of high molecular weight polyaromatic hydrocarbons in soil. *V. sinuatum* has morpho-functional suitable features for developing a dynamic and efficient rhizospheric network. In fact, it showed a great adaptability to interact with the chosen microbial consortium. Endophytic relationships and rhizospheric dynamics are already manifest at 60 days for infection and are rapidly consolidated with the development of an extended and effective mycelium. The choice of a microbial consortium consisting of selected mycorrhizae and PGPR bacteria with site-specific relationships may have resulted in molecular signals (mainly hormonal) pathways of *V. sinuatum* roots. The symbiotic relationships between fungi and plant roots (mycorrhizae) deserve special attention because they can increase tolerance of plant species involved in phytoremediation [71], [72], [73] and enhance PAHs rhizodegradation. It has been widely recognized that, under stress, mycorrhizal plants had higher enzymatic antioxidant activity than non-mycorrhizal plants, since AM fungi intensify their production to (i) alleviate of ROS breakage protecting the hosting plant and (ii) help the AM plants to enhance their stress tolerance [30,74].

This system of rhizoplane improvement let the plant to overcome adverse environmental conditions and also led to a substantial increase in the overall phenotype fitness [75]. *V. sinuatum*, together with our consortium, showed a significant adaptability in terms of redox biology. In fact, our results about antioxidants and stress markers showed that the plant is able to adapt its basal metabolic levels towards a new redox level at about between the 120-240 days period. The lower stress could be related to PAHs degradation that progressively increased, resulting in minor level of abiotic stress for plants. In addition, the removal of PAHs, which are hydrophobic and are primarily attached to soil particles, may have enhanced soil water-holding capacity [76] and consequently the accessible water for plants. In addition, the co-activity of plant and associated microorganisms enhances nutrients availability in soil [77], with direct effect on bio-fortification of plants.

Our research indicates that the excellent rhizospheric environment determined by *Verbascum* in association with the microbial consortium constitute a powerful tool for rhizoremediation. The role of plant uptake was negligible. However, the uptake of secondary metabolites produced by the co-metabolism was not investigated although suggested to be relevant [78]. It is well-known that vegetation is expected to promote biodegradation since the plant root zone (rhizosphere) has a more abundant microflora than soils without plant cover [79,80]. In addition, plants can altering root exudation exerting selective and promoting effects on specific microbial populations in relation to the environmental conditions [81]. The great degradation of 4, 5 and 6 rings PAHs highlights the proliferation of a rhizospheric environment particularly oriented towards the aggression of these harmful and recalcitrant xenobiotics. As matter of fact a substantial increase of degrading genes, especially dehydrogenases and laccases, was evident. The use of a saprophytic fungus, *Pleurotus ostreatus*, as an additional element of the rhizodegradation consortium was based on the assumption that its enzymes (mainly laccases) are able to oxidate high recalcitrant PAHs [82]. Furthermore, a consolidated associations between plant and microorganisms was evidenced by the increase of the rhizosphere soil enzymatic activity. Indeed, plant roots are able to stimulate the soil enzymatic activities by creating favorable conditions for microbial activities [83]. This association also enhance the transformation and/or degradation of pollutants and it presumably result in higher amounts of carbon source in the soil system, which may further led to rising soil enzyme activity [42].

As regards the degradation kinetics we obviously assumed different dynamics in the reduction of PAHs and surprisingly found also a strong aggression of the most recalcitrant PAHs (5 and 6 rings). Overall, the proposed biotechnology highlight a great degradation performance which decrease as levels of pollutants increase.

The great performance of the proposed biotechnological combination is clearly showed by the results about PAHs concentration levels, the enzymes activities and degrading genes in soil.

The *V. sinuatum* has proved to be a valid genotype for the rhizosphere engineering as it (i) well adapted to disturbed systems, (ii) quickly and effectively accepted the added microbial consortia and (iii) directly and indirectly participated to the creation of a radical environment favorable the rapid degradation of recalcitrant compounds such as PAHs. Furthermore, its ability to adapt to competitive systems even with saprophytic fungi make it particularly suitable for degradation processes of organic xenobiotics at the rhizosphere level.

## 5 Conclusion

To our knowledge, this is the first study which analyze the *Verbascum sinuatum L.* and microbial consortium (composed of mycorrhizae and bacteria) association to exploit its biodegrading traits in the presence of PAHs contaminated soil. Our findings contribute to progress in in-situ bioremediation research topic by analyzing at a multiscale level the plant environment where biodegradation occurs. In fact, the PAHs removal is mainly explained by the enhanced microbial degradation patways. However, *Verbascum sinuatum* L. root exudates had an important role in the selection and the growth of rhizosphere microbes and directly and indirectly co-partecipate to PAHs solubility and bioavailability in soil, thus promoting biodegradation.

This mesocosm experiment also demonstrates the positive role of microrganisms in promoting alleviating oxidative stress in *V. sinuatum* exposed to environmental stress, such as highly polluted soil. Root mycorrhizal colonization induced a higher plant defense ability leading to plant enhanced stress tolerance. Ultimately, the extension of rhizospheric bioactivity was the results of a successful metaorganism for restoring balance within the soil ecosystem under PAHs contamination. The combined biological systems used for the removal of PAHs in soils demonstrated to have great prospects, which at long last manage environmental and human health protection through relatively low expensive solutions. There is still no known general remediation approach since the remediation technology is strictly site-specific, but more efforts under a sustainable perspective would be need. In this context, researching the direct effects of pollutants on the roots exudates release, could surely lead to obtain new insights on plants ability in the development of associated soil microflora, which is of primarly importance in the degradation of high recalcitrant compounds.

## Declaration of Competing Interest

None

## Appendix Supplementary materials

Image, application 1
