# Supplementary material for: The remediation potential for PAHs of Verbascum sinuatum L. combined with an enhanced rhizosphere landscape: A full-scale mesocosm experiment
Source: Biotechnol Rep (Amst). 2021 Jun 27;31:e00657. doi: 10.1016/j.btre.2021.e00657 (PMC8264111; doi:10.1016/j.btre.2021.e00657)
Supplement: Supplementary file 1 [file mmc1.docx]

**Supplementary Materials for**

**The remediation potential for PAHs of *Verbascum sinuatum* L. combined with an enhanced rhizosphere landscape: a full-scale mesocosm experiment.**

Daniela Zuzolo, Rosaria Sciarrillo, Alessia Postiglione, Carmine Guarino*

*Department of Science and Technology, University of Sannio, via de Sanctis, 82100 Benevento, Italy*

*Corresponding Author (CG): guarino@unisannio.it

Table S1 – Main physico-chemical features of mesocosms soil.

| **Area** | **pH** | **CEC** | **Clay** | **Silt** | **Sand** | **TOC** |
| --- | --- | --- | --- | --- | --- | --- |
|  |  | meq/100 g | % | % | % |  |
| A3 | 7.5 | 27 | 3.2 | 28.2 | 26.3 | 21481 |
| A4 | 8.1 | 21 | 3.6 | 32.7 | 17.2 | 5067 |
| A6 | 6.3 | 14 | 5.3 | 47.0 | 24.2 | 3858 |

Table S2 – Concentration (mg/kg) of PAHs congeners in roots of mesocosm-grown *Verbascum sinuatum* L.

| Plant material | Growth Area | Pyr | BaA | Chr | BaP | BbF | DahA | BkF | BghiP | DaeP | DahP | DaiP | DalP | IP |
| --- | --- | --- | --- | --- | --- | --- | --- | --- | --- | --- | --- | --- | --- | --- |
| Root | A3 | 1.2 | 0.7 | 0.9 | 0.8 | 1.0 | 0.1 | 0.4 | 0.6 | 0.1 | 0.2 | 0.1 | 0.2 | 0.6 |
| Root | A4 | < 0.1 | < 0.1 | < 0.1 | < 0.1 | 0.131 | < 0.1 | < 0.1 | < 0.1 | < 0.1 | < 0.1 | < 0.1 | < 0.1 | < 0.1 |
| Root | A6 | < 0.1 | < 0.1 | < 0.1 | < 0.1 | 0.13 | < 0.1 | < 0.1 | < 0.1 | < 0.1 | < 0.1 | < 0.1 | < 0.1 | < 0.1 |

Table S3 – Concentration (mg/kg) of PAHs congeners in leaves of mesocosm-grown *Verbascum sinuatum* L.

| Plant material | Growth Area | Pyr | BaA | Chr | BaP | BbF | DahA | BkF | BghiP | DaeP | DahP | DaiP | DalP | IP |
| --- | --- | --- | --- | --- | --- | --- | --- | --- | --- | --- | --- | --- | --- | --- |
| Leaf | A3 | < 0.1 | < 0.1 | < 0.1 | < 0.1 | < 0.1 | < 0.1 | < 0.1 | < 0.1 | < 0.1 | < 0.1 | < 0.1 | < 0.1 | < 0.1 |
| Leaf | A4 | < 0.1 | < 0.1 | < 0.1 | < 0.1 | < 0.1 | < 0.1 | < 0.1 | < 0.1 | < 0.1 | < 0.1 | < 0.1 | < 0.1 | < 0.1 |
| Leaf | A6 | < 0.1 | < 0.1 | < 0.1 | < 0.1 | < 0.1 | < 0.1 | < 0.1 | < 0.1 | < 0.1 | < 0.1 | < 0.1 | < 0.1 | < 0.1 |

Table S4 - Arbuscular colonization (AC), vesicular colonization (VC) and hyphal colonization (HC) *Verbascum sinuatum* L. roots

| **Sample** | **Description** | **% AC** | **% VC** | **% HC** |
| --- | --- | --- | --- | --- |
| *1* | *Verbascum sinuatum* L. (in-situ) | 2.7 (0.9) | 5.9 (1.1) | 21.3 (1.3) |
| *2* | *Verbascum sinuatum* L. (mesocosm) - T_1_ | 3.4 (1.3) | 6.6 (1.2) | 26.1 (1.5) |
| *3* | *Verbascum sinuatum* L. (mesocosm) - T_F_ | 12.1 (1) | 16.5 (1.5) | 49.7 (0.9) |

Data are means (samples= 3) with standard deviations in brackets. 1 correspond to *Verbascum sinuatum* L. grown in-situ; 2 and 3 correspond to *Verbascum sinuatum* L. grown in mesocosm pots with a microbial blend (Consortium Bagnoli 2018@”) and *Pleurotus* *ostreatus*, sampled at T_1_ and T_F_ experimental times, respectively.
